# A Systematic Review on Professional Regulation and Credentialing of Public Health Workforce

**DOI:** 10.3390/ijerph20054101

**Published:** 2023-02-24

**Authors:** Olga Gershuni, Jason M. Orr, Abby Vogel, Kyeongki Park, Jonathon P. Leider, Beth A. Resnick, Katarzyna Czabanowska

**Affiliations:** 1 Department of International Health, Care and Public Health Research Institute (CAPHRI), University of Maastricht, 6229 GT Maastricht, The Netherlands; 2 Department of People and Health Studies, Fontys University of Applied Sciences, 5631 BN Eindhoven, The Netherlands; 3 Center for Public Health Systems, School of Public Health, University of Minnesota, Minneapolis, MN 55455, USA; 4 Center for Public Health Systems and Division of Health Policy and Management, School of Public Health, University of Minnesota, Minneapolis, MN 55455, USA; 5 Department of Health Policy and Management, Johns Hopkins University, Baltimore, MD 21205, USA

**Keywords:** public health workforce, professional credentialing, professional regulation, systematic review

## Abstract

The public health workforce (PHW) counts a great variety of professionals, and how services are delivered differs in every country. The complexity and the diversity of PHW professions also reflect structural problems of supply and demand of PHW in various organizations and health care systems. Therefore, credentialing, regulation, and formal recognition are essential for a competent and responsive PHW to address public health challenges. To ensure comparability of the credentialing and regulation systems for the PHW and to enable its collective action at the macro level in the event of a health crisis, we systematically analyzed documented evidence on the PHW. A systematic review was selected to answer the research questions: (1) what are the most effective aspects and characteristics in identified programs (standards or activities) in professional credentialing and regulation of the PHW and (2) what are common evidence-based aspects and characteristics for the performance standards to support a qualified and competent PHW? The identification of professional credentialing systems and available practices of the PHW was performed systematically using a systematic review of international resources in the specialized literature published in English. The PRISMA framework was used to verify the reporting of combined findings from three databases: Google Scholar (GS), PubMed (PM), and Web of Science (WoS). The original search covered the period from 2000 until 2022. Out of 4839 citations based on the initial search, 71 publications were included in our review. Most of the studies were conducted in the US, UK, New Zealand, Canada, and Australia; one study was conducted in an international context for professional credentialing and regulation of the PHW. The review presents specific professional regulation and credentialing approaches without favoring one of the proposed methods. Our review was limited to articles focused on professional credentialing and regulation of the PHW in the specialized literature published in English and did not include a review of primary PHW development sources from international organizations. The process and requirements are unique processes displaying knowledge, competencies, and expertise, regardless of the field of practice. Continuous education, self-regulatory, and evidence-based approach can be seen as common characteristics for the performance standards on both community and national levels. Certification and regulation standards should be based on competencies that are currently used in practice. Therefore, answering questions about what criteria would be used, what is the process operation, what educational background the candidate should have, re-examination, and training are essential for a competent and responsive PHW and could stimulate the motivation of the PHW.

## 1. Introduction

The 2000s have proven particularly challenging for the frontline public health workforce (PHW). Generational retirement associated with baby boomers aging out of the workforce and job losses from the post-2008 “Great Recession” in the United States and Europe have made recognition of the PHW of utmost necessity. A competent, healthy, and satisfied PHW is critical for quality delivery of care, especially in times of crisis when the provision of public health (PH) services is a challenge, as demonstrated by conflict in Ukraine [1]. Despite this need, the PHW shortage persists and needs to be addressed.

Prior works have illustrated substantial shortcomings, such as a scarcity of evidence-based research contributing to “a fundamental challenge to the field of public health workforce development…” [2]. PH practice focuses on the population’s collective responsibility in tackling health-related issues and inequalities, identifying risks, and improving societal well-being [3]. Developing qualified and competent PHW staff may require navigating arcane and disparate regulations, registrations, and certifications that differ dramatically between jurisdictions. The PHW is on the frontlines of society, yet it lacked the ability to prevent or mitigate the COVID-19 pandemic, a tragic example of what happens when society neglects the critical role of the PHW. PH organizations dealing with pandemic consequences are responsible for different tasks, from proper hand hygiene to risk communication and building trust on community and country levels [4]. The assurance of effective and timely response to PH challenges, including the COVID-19 pandemic, requires a high-quality, educated, and, ideally, certified PHW, depending on role and responsibility.

However, it is evident that a sustainable PHW needs guidelines and solid standards to guarantee professional satisfaction and mobility [5]. This means that regulatory authorities and responsible organizations cannot underestimate the importance of a clear assessment of the qualifications of a PHW needed to provide essential public health services. This process requires systematically documented evidence of education, training, and professional experience. “Professionalism”, “professionalization”, and “professional” are all terms with a core component: *a profession*. Professional groups share the same standards-based skills and knowledge. The requisite skills and expertise differ by profession, generally defined and managed by a commonly accepted authority that also establishes required trainings and verification processes [6]. The International Standard Classification of Occupations report presents an extensive overview of health-related occupations; although such an overview allows grouping the professions, the comparability in functioning and performance of practice varies per country. Additionally, (major) subgroups consist of minor groups with specific skillsets and responsibilities [7]. Trust in quality of care and, thus, public confidence can be achieved by introducing professional and ethical codes of conduct; however, the field of public health encompasses many disciplines—especially when considering a “wider public health workforce”—that have widely varying professional regulation and certification processes [8,9]. This complexity is compounded when considering international context, as each country’s definition and use of “public health workforce” differs slightly, as do regulation, licensure, and certification considerations [9]. This paper surveys these considerations.

### 1.1. Public Health and Public Health Workforce

The term “public health” can be used and applied differently depending on the country or setting, context, governance, and other factors, such as financing and distribution. Further, there are variations in expected performance, preparation, professional competencies, and standards for PH professionals [10]. Despite being of strategic importance to international health and valuable to the global economy, the scope of the PHW is poorly and inconsistently defined [11]. The definition and composition of the PHW is unique in every country and applies to individuals who are “engaged primarily in improving the health of populations” [10]. Often conflated with clinical health personnel—public health professionals are often referred to as “healthcare workers”—thus, the non-clinical PHW suffers from a lack of recognition and the accompanying harms of such a lack, including limited postgraduate opportunities, opaque career paths, and inconsistent application of professional regulation. Although PH is “everybody’s business,” the variety of roles and definitions within PH confuse a clear definition and responsibility. For example, one common perspective defines the “core” PHW as persons engaged in public health activities or provision of public health services who “identify public health as being the primary part of their role” [12,13]. There exists a broader definition of the PHW, the “wider” PHW, as “any individual who is not a specialist or practitioner in public health, but has the opportunity or ability to positively impact health and wellbeing through their (paid or unpaid) work” [14].The varied definitions for the PHW suggest a lack of accountability and a need for harmonization.

As a practical matter, the differences between health systems and terminology make professional roles and organizational responsibilities difficult to compare. Such problems are associated with enumeration and lack of suitable taxonomies. The importance of a sustainable PHW is not always reflected by the baseline demand for services—exemplified with the emergence of the COVID-19 pandemic [15]. In addition, credentialing, regulation, and formal recognition are essential for a competent and responsive PHW [16]; these aspects are today, generally, based on “good-faith expectations for improving individual and organizational performance” rather than systematized practice or knowledge requirements [17]. Further investigation is needed to understand how professional credentialing and regulation intersect with the public health workforce.

### 1.2. Professional Credentialing and Regulation of Public Health Workforce

A source of misunderstanding among professional practitioners and academics may be within “the interplay among the concepts of competencies, credentialing, and accreditation [in public health]” [16]. Key terms for this study (“professional credentialing”, “professional regulation”, “certification”, and “registration”) should be defined within a PH context. Professional credentialing, by one definition, is “the process of obtaining, verifying, and assessing the qualifications of public health professionals to provide services/operations for a public health organization, institution or agency” [18]. Another definition describes it as a process “to assign specific clinical responsibilities to health practitioners based on their education and training, qualifications, experience and fitness to practice within a defined context” [16]. Professional regulation (including licensing, certification, and registration), on the other hand, largely depends on a country’s context, wherein a varied combination of PH professions are governed by statutory regulation [16]. Such regulation, where present, can assure baseline professional skill sets, a continuity of essential service knowledge, and appropriate staff placements. Certification is distinguished from licensure by its nongovernmental and voluntary nature [16], and is “a process by which an authorized body/agency, such as a professional body or governmental agency, grants recognition to those individuals as having met certain predetermined requirements or criteria” [19]. Registration, in contrast, requires categorical organizational enumeration of the PHW and is not always criteria-based. Finally, licensure can be obtained by a responsible body granting a professional license to perform work activities [16].

Systematizing regulation and credentialing processes may benefit the development of a competent and responsive PHW. Various sources discuss the challenges and benefits of professional recognition for PH professionals, including PH nurses, PH nutritionists, and PH administrators [20,21,22,23,24]. The organizations responsible for PHW training are sometimes accredited institutions; for example, in the US, the Council on Education for Public Health (CEPH) is responsible for accreditation procedures for higher education in public health. The graduates of accredited PH schools and programs have the option to undergo credentialing processes as one of the trajectories for successful professional recognition [2]. In the UK, professional healthcare regulation is divided into nine statutory health and care regulators (such as the Nursing and Midwifery Council (NMC) and the Health and Care Professions Council (HCPC)) [25]. These regulators have a dual function of both protecting the public and maintaining professional standards. The Professional Standards Authority supervises the regulation for Health and Social Care, a so-called “super-regulator” [25]. New Zealand developed The Credentialing Framework for Health Professionals, where seven principles are applied to all health professions (including PH) [26]. The credentialing process in Australia is state-based, focused on matching the skills and experience on clinical needs, and provides recognition of various professions [27].

Although some countries have developed structured and systematic approaches for credentialing of some core public health and related professions [13,16,17,24], credentialing and regulation in the PHW as a whole remains spotty. This varied patchwork of credentialing and regulatory processes may present a source of confusion for those entering the workforce and employers. Therefore, addressing the issue of PHW credentialing and regulation is critical to mounting an adequate response to PH threats, such as disease outbreak, which do not respect governance borders.

### 1.3. Study Aim and Review Questions

The COVID-19 pandemic had a massive impact on governmental public health and healthcare, those persons tasked with responding to the global health emergency. The stressors on the public and private systems have made clear that appropriate professional regulation and credentialing must be in place to support the core and wider PHW in fulfilling specific competencies and ensuring safe and adequate service delivery. This systematic review attempts to identify salient themes for professional regulation and credentialing of the PHW, performance standards to advance regulatory sophistication, and other topics related to the development of a competent PHW. More specifically, we investigate the following research sub-questions:

1. What are the most effective aspects and characteristics in identified programs (standards or activities) in professional credentialing and regulation of the public health workforce?
2. What are common evidence-based aspects and characteristics for the performance standards to support a qualified and competent public health workforce?

## 2. Materials and Methods

A systematic review was selected to answer the research questions. The review was conducted according to the PRISMA statement, which provided a framework for a systematized approach toward document identification and review [28]. The systematic review protocol was registered with PROSPERO (CRD42022301269). Revised methods for that protocol and those used in this study are available as a supplementary file (Protocol S1). No research involving human subjects was performed in this study.

### 2.1. Search Strategy

The search strategy aimed to obtain original peer-reviewed literature, commentaries, analytic essays, and gray literature that addressed the research question. Articles were all obtained using “Publish or Perish 8” (PoP) software to record and conduct replicable queries through Google Scholar (GS), PubMed (PM), and Web of Science (WoS) databases [29]. The combination of databases is often used in the area of PH to appraise all available scientific resources in this field. Keyword queries each included multiple search terms from each domain to return potentially relevant articles from all databases, with queries carried out 10–11 January 2022 (presented in full in Dataset S2).

### 2.2. Study Selection

There were no restrictions on study design, country of performance, or outcomes measured; only articles in English and published within the years 2000–2021 were considered. Study selection followed a two-phase process; first, performing screening and eligibility, and then, critical appraisal; each stage had two independent reviewers and a third to resolve conflicts. First, citation titles and abstracts were screened for relevance to the research question and then for eligibility with either of the sub-questions. Eligible articles related to PH and the PHW and described professional credentialing or professional regulation (including professional registration, licensing, and certification); however, our review was limited to widely available specialized literature in English. Exclusion criteria included subjects focused within the public health education domain such as approaches for teaching, training, or academic programs (i.e., activities preceding professional processes) and if the material type were not likely to provide substantial content (e.g., conference abstracts). For the second stage of review, full text materials were obtained for each retained eligible citation and critically appraised using a respective critical appraisal tool made available by the Joanna Briggs Institute [30], such as the “Checklist for Analytical Cross-Sectional Studies”.

### 2.3. Data Extraction and Key Themes

Metadata fields from the PoP citation output (e.g., author, year) were included for all citations. More fields were added to track screening and eligibility dispositions as well as for data extraction. Summarization and theming were performed on each included material. For thematic analysis, occupations and countries represented in each material were extracted to develop unique counts of each. Due to the heterogeneous nature of materials and likelihood that multiple different occupations or different countries may be included, certain studies may represent multiple relevant items. We then analyzed findings descriptively and reported them within tables and narrative. Due to the heterogeneous and primarily qualitative nature of materials, the authors decided to forego meta-analysis of study outcomes.

## 3. Results

A total of 4839 citations were initially obtained from the broad search strategy. Of those, 1233 duplicates were identified and excluded, leaving a base of 3606 citations. Screening (titles and abstracts) against the research question and sub-questions led to further attrition. Primary reasons for removal at screening were due to the abstract or title not explicitly relating to the PHW or not substantially relating to credentialing, certification, regulation, etc. We excluded the articles presenting the PH education domain or undergraduate degree, those related to PHW education and training on various levels (master’s and bachelor programs), evaluations or teaching approaches in a field of PH, assessment or teaching approaches in an area of PH, those articles in which the subject was credentialing but that only described the evidence of a completion of a particular course, and conference papers.

**Figure 1:**
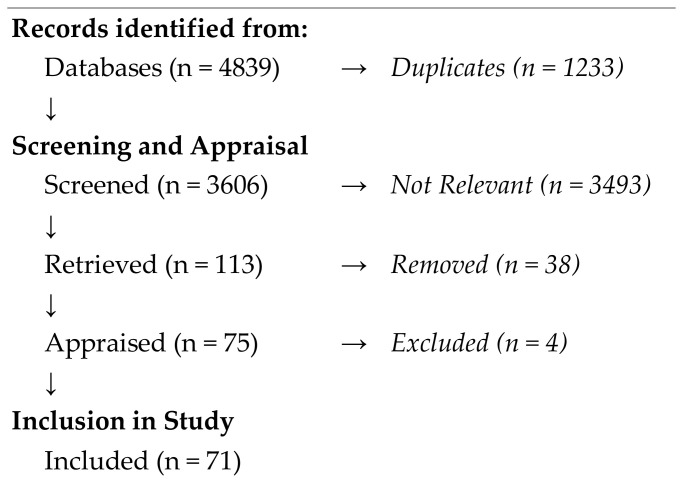
PRISMA diagram for study.

Following screening, 38 citations were removed due to inaccessibility (e.g., we could not find the full text, or the article was not in English). Next, the 75 remaining full-text materials were critically appraised; 4 materials were excluded within critical appraisal. Finally, 71 articles were retained for our study (Figure 1).

### 3.1. Occupations Represented

The professions discussed in the specialized literature and selected for the analysis are reflected in our systematic review (Table 1). Our analysis shows that 23 studies were related to the field of nursing, which included midwifery. The next largest portion of the analysis concerned public health. These studies were limited to general traditional public health concepts which included public health specialists, health educators, public health nurses, epidemiologists, sanitarians, public health informaticians, program specialists (e.g., environmental health, public health emergency preparedness, maternal and child health), public health laboratorians, childcare licensing surveyors, public health social workers, and dietitians. We separately categorized wider public health that discusses health trainers, allied health professionals, educators (e.g., K-12, college), midwives, pharmacists, dentists, dental hygienists, health informaticians, opticians, physicians, clinical nurses (e.g., general practice, district, school, occupational health), clinical laboratorians, veterinarians, health or fitness instructors, clinical social workers, environmental specialists (e.g., engineers, urban planners, toxicologists, hydrologists), lactation consultants, poison control, medical examiners or coroners, emergency managers, industrial hygienists, behavioral health providers (e.g., counselors, prevention specialists), and health facility surveyors. The included studies also covered fields in health education, dentistry, social services, and nutrition (Table 1).

### 3.2. Countries Represented

**Figure 2:**
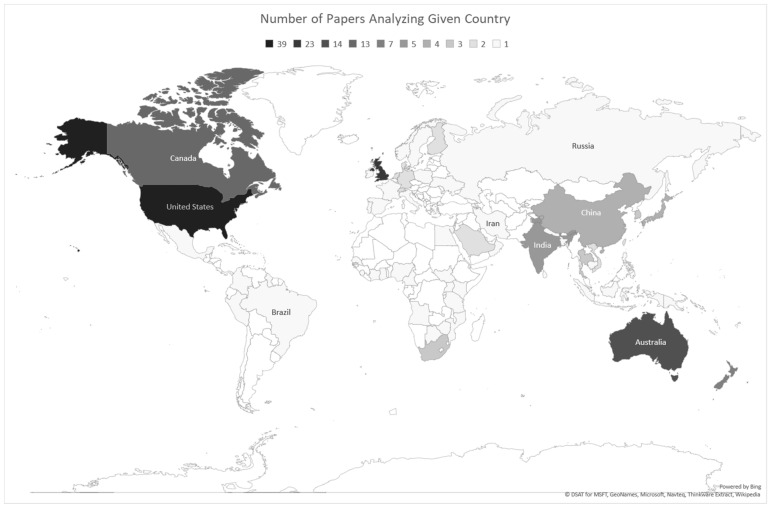
Countries included within articles.

Only countries discussed in the specialized literature were reflected in our systematic review (Figure 2). The articles which met criteria for analysis (see Section 3.1) skewed heavily toward English-speaking countries: the United States, the United Kingdom, Australia, Canada, and New Zealand, respectively, represented the top five countries by frequency of analysis. Information for the regions of Eastern Europe, Central Asia, and sub-Saharan Africa was particularly sparse, and the quality of analysis for other, more rarely analyzed regions, contained less detail than for those English-speaking countries previously mentioned.

For convenience, we use the convention of a four-digit identifier (e.g., “0048”) when referencing findings from our review, as opposed to the journal’s citation style, with identifiers found in the first column of Table 2. The full references for four-digit identifiers are included in the dataset file (Dataset S2) and not displayed in the references list due to a large amount of the included materials.

### 3.3. Key Findings for Credentialing

Although in some PH professions and countries, credentialing is a strict requirement for nurse practitioners (0638) or internationally trained nurses (0048), it can be voluntary and not prerequisite for a position (0016, 0226). To maintain credentialing, continuing education is required (0033, 0067, 0226); it varies in terms of the number of hours and years required. Regardless of the system, professionals must meet specific predefined practice standards (0638, 0473, 0388). Examination, as a measurement, validates a process, is a reliable source (0550, 0501, 0126, 0083), and can be replaced by portfolio-based assessment (0027); it is an absolute requirement for the employment of internationally educated nurses (0148). Credentialing seems to become stricter, involving revisions when direct patient care, an international component, or an acute care setting is mentioned (1690, 0638, 0048). A degree/educational background is a must (1690, 0550), although, in some cases, accurate documentation of experience in the field is added to the credentialing process (1690, 0550, 0016). Credentialing status can be monitored and directly influences the perception of competence in knowledge and skills (0033, 0501, 1690, 0083). Professional development was mentioned frequently as one of the benefits of credentialed professionals (0473, 0145, 0096). A complete overview of the data is provided in Table 2.

### 3.4. Key Findings for Regulation

A main finding of the review was the scopes and varieties of regulatory frameworks and related standards for different public health practitioners (see Table 1). There were many examples in the literature of how some practices are highly regulated by governments to assure critical aspects of service delivery, such as quality, timeliness, and access (0017, 0024, 0052, 0095, 0116, 0377, 0388, 0445, 0473, 0540, 0748, 1690, 4236). Regulations were described as processes and procedures to ensure “fitness to practice” (0325, 0445, 0494) to deliver limited “scopes of practice” for enumerated sets of procedures and services (0513). Regulatory authorities may set forth specific frameworks or standards that specify conduct, education, training, continuing professional development, disciplinary processes, and other important regulatory features to ensure quality of services (0052, 0055, 0078, 0087, 0253). However, regulation does not always lead to efficient or effective delivery of services (0085), though some efforts are dedicated toward improvement of regulatory practices (0335, 0377, 0451).

Regulated professions are often monitored through a public or private registry (0024, 0052, 0060, 0077, 0109, 0276, 0460, 0473, 0561, 0667) that may include oversight by a variety of entities (0074, 0078, 0276). Primary public health registries/registers include:

- Emergency System for Advance Registration of Volunteer Health Professionals (ESAR-VHP, US);
- General Medical Council (GMC, UK);
- Nursing and Midwifery Board of Australia (NMBA);
- Nursing and Midwifery Council (NMC, UK);
- UK Public Health Register (UKPHR);
- UK Voluntary Registrar for Public Health Specialist (UKVR).

Regulations incorporated in this paper’s discourse were limited to health care services delivery and primarily related to the nursing and midwifery professions (0017, 0052, 0055, 0077, 0109, 0325, 0395, 0513, 0667, 0867, 0964, 4236) as well as to cognitive behavioral therapists (0116) and physician assistants (1452). A complete overview of the data is provided in Table 2.

### 3.5. Key Findings for Certification

Certification is available for individuals associated with the autonomy of practice, professionalism, professional development, and strengthened professional preparation (0005, 0149, 0473, 0501). Maintaining certification: renewal of the certificate varying in years and providing proof of continuing education credits (0029, 0067, 0126, 0129, 0443, 0501, 1452) requires an administrative body to implement a process for verifying that an individual is qualified (based on skills or experience) for their roles or duties. It may also involve training, assessment, and ongoing continuing education (0094). The eligibility requirements may also include a (re)certification examination or renewal of certification (0388, 0443, 0473, 0494, 0501,1452, 1552). The examination may include core and cross-cutting areas (0501), holding an active license, having appropriate education, and demonstrating knowledge and experience in the specialty field (e.g., clinical examination or a workplace assessment) (0016, 0443, 0494, 4648). Additionally, attendance and participation in professional events and activities, earning other related certifications, and following courses are related to certification (0501). A complete overview of the data is provided in Table 2.

### 3.6. Key Findings for Registration and Licensure

Registration can be provisional or clinical competencies-related (specialty), and the qualified professional can apply for it after a certification procedure (0494). However, registration examination (clinical, written) is required (0494, 0667) after completion of the education (0667); the concept of “(un)fitness to practice” can be a requirement when a professional seeks registration. There are registration requirements such as engagement in continuing professional development (0060). Additionally, the professionals seeking (re)registration need to present evidence of experience regarding the improvement of professional practice (0060) and acknowledge their status and skills (0451). The reregistration varies in terms of time intervals, and its validity differs (e.g., five years) (0060, 0067). The registration process involves professional standards, skills, and competencies (0473, 0095), and it can be “limited” when the supervision is mandatory (0494). Registration supports public protection and is responsive to complexity (0451). Registration systems to monitor the active workforce are scarce (0460). In some countries, a professional can reregister, ensuring that specific qualifications are met before medical professionals can begin practicing (0460). Additionally, some professionals (e.g., nursing) require certification or passing an exam before registration (0667). Additionally, new ways to register the volunteering workforce are in place, aiming to deliver a coordinated and collaborative response (0090).

Regulation through licensure sets standards for education, clinical training, and governmental oversight and accountability (0052). Some countries require an examination before issuing a license; others provide it after graduation for a fee (0460). A written exam is required (0067). In addition, a set number of hours are required annually or biannually to renew the license (0443). The government regulates certification and licensing processes (1017). However, when it comes to transition programs for international professionals, various categories are in place such as preregistration, pre-hire programs, and post-licensure post-hire programs (0436). A complete overview of the data is provided in Table 2.

### 3.7. Key Findings with Respect to COVID-19

Only five articles included in this systematic review were published after the outbreak of COVID-19, and only two of those five discussed the pandemic’s effect on the PHW. The authors, analyzing scopes of practice in Canada, Australia, and the UK, advocated for “optimizing the workforce by ensuring all professionals are practicing to full scope”, pointing out that expanding the scope of PHW professions and increasing flexibility in scope were key elements of the response to COVID-19 (0074). Addressing licensure regulation, the same authors suggest that “regulatory frameworks need to be made nimbler and more responsive” to better support adaptation in the face of rapidly evolving health care needs. One source discusses data from lower- and middle-income countries (0096) and the invaluable role of health support workers and community health workers in combating the pandemic where access to professional care is less widespread and regulatory systems remain nascent.

### 3.8. Recommendations from Materials

The criteria for certification for PH professional should be based on the standards and the competencies (currently used and one under development) (0005, 0126). Stimulation of lifelong learning, leadership, and workforce development are enhancing the recognition of the profession (0126, 0094). Additionally, it should be clear how the process is operated and financially supported (0016, 1452, 0094), whose responsibility it is (0443), and what and how to apply the criteria for certification (0016,0029). Additionally, it is suggested to investigate what the impacts of the technique are on affected person outcomes, quality of care, and the possibilities for curriculum development (1039, 1452).

Credentialing process requirements should be simplified, standardized, and widely accepted, thus stimulating the PHW to take this path (0033, 0126, 0048). The establishment and continuous development of common core competencies (0028, 0092) and active community participation will stimulate effective collaboration in the workplace, increasing value and encouraging employees’ personal development (0048, 0083, 0067). Employers should participate in reducing the burdens for credentialing, such as financial contribution, added value in terms of employment, and clarification of job responsibilities (0129, 0145, 0067). The ecological all-inclusive competency model, with a voluntary nature, could be a key to the fair contribution of the PHW to meet credentialing qualifications (0054, 0067, 0082, 0092).

Regulatory bodies and frameworks must specify essential functions: setting the scope of practice, (pre)registration, education, relicensing code of conduct and ethics (0078). A clear description and functions will stimulate professionals to develop new professional pathways (0095, 0078). In addition, cooperation, coordination, and sharing of good practices could be improved for global regulatory practices (0096). A responsive register is seen as a mechanism that addresses reality and is up to date (0090, 0377, 0388). To ensure the regulation process, PH job description, competencies overview, assessment, development, and application of it to the actual job must be disclosed (0017, 0055). There is a need for a clear assessment of the knowledge and skills of professionals (0748) and the development of a precise standard for the non-medical and wider PHW (0060). Such criteria for PH practice include legislation, education, recruitment, supervision, and guiding principles (0116). However, the regulation development must be based on joint agreement and staff involvement at every stage (0052, 0055). In addition, other essential aspects, such as interprofessional learning (0060), responsibility empowerment, and career guidance (0451) are needed to address the recruitment and development of future PH professionals within the local context (0085). A complete overview of the data is provided in Table 2.

## 4. Discussion

The purpose of this study was to review evidence-based literature on professional credentialing and regulation programs, standards, and activities for the PHW. We synthesized several categories, such as country of performance, the field of PH (organization or profession), evidence-based approach, the methodological background of the performance standards, and transferability (see Dataset S2). The field of PH remains challenging in terms of recognition and definition. In our systematic review, we have not limited ourselves to certain professions; instead, we considered the core and wider PHW. In terms of credentialing and regulatory processes, the authors summarized the procedures and specific requirements. Additionally, we attempted to answer multiple questions in key subject areas:

- Mandatory vs. voluntary nature of regulation, certification, or credentialing;
- Governance of regulation, certification, or credentialing (e.g., who verifies requirements, public or private governance);
- Specific mechanisms and functions of regulation, certification, or credentialing (e.g., the presence of a registry, academic or credential requirements).

Our systematic review presents an overview of the most compelling aspects and characteristics in identified professional credentialing and regulation standards in the PHW. However, this review is reflected only in specialized literature and, thus, does not take into account organizational or national resources. The selected resources highlight the common evidence-based aspects and attributes for the performance standards to support a qualified and competent PHW. The organizations responsible for credentialing underline the importance of credentialing for the PH workforce. The specificity of the profession, regulation, and credentialing programs, and the applicability of the results to other professions could be problematic at first glance. However, a clear description of essential characteristics, performance standards, and competencies should stimulate the consensus towards uniformity and structure, leading to certification, credentialing, regulation, and registration of the PHW.

Furthermore, the inconsistency in the definitions used in the studies demonstrates the complexity of the concept of credentialing, regulation, registration, and certification. Although we can only assume, due to the limited amount of information analyzed in this review, it also shows that evidence needs to be built consistently to assure accuracy. Despite the discussions about definitions and previous contributions to a shared concept, the concepts remain vague and are used interchangeably. According to Hoard and Tosatto, credentials are an individual’s professional qualification, which may include both licensure and board certification [31]. The most common justifications for certification are the protection of public interest, creating general knowledge about a profession, lifelong learning for certified individuals, distinguishing themselves from noncertified peers, and to help employers make hiring decisions [32].

The variety of PH systems across countries with an unblended organizational structure, history, and population expectations, with Illinois and Ohio as an example from the US, preclude generalization of findings even within one country [21,33]. Another example from the EU can ensure instead an “automatic recognition” procedure for PH professionals registered with a regulatory body in any of the member state countries; such allowance is based on meeting the professional requirements and performance competencies [34]. The European study proposed an individual, voluntary practitioner-based certification model for professional recognition [20]. One review study within an international context performed an overview of available studies linking certification or credentialing in the PHW. However, the individual and organizational performance of the process raises concerns because of the differences in investment in the PHW, expectations, and characteristics of responsible organizations and agencies [17]. Another good example of registration is maintaining its “fitness to practice”, which is the combination of the competencies, appropriate professional behavior, and attributes required for the need to be in place to perform the services effectively and safely; professional registration as a health practitioner relies on maintaining its “fitness to practice” aspects to match the required competencies, skills, and knowledge to the performance quality [35,36].

Effective PH practice requires a focus on collective responsibility of the population in terms of tackling health-related issues, inequalities, risk identification and societal well-being [37]. The PHW is constantly urged to make decisions under conditions of uncertainty, interact with different audiences, and to be flexible according to population-based health principles and priorities. The PHW is dramatically faced with disruptive reality, with the COVID 19 pandemic as a tragic example of the key role of the PHW, day in and day out [38]. PH organizations, which dealt with pandemic consequences, are responsible for different tasks, from proper hand hygiene to risk communication and building trust on community and country levels [16].

COVID-19 challenged international, national, and subnational PH systems during its spread [39]. The pandemic exposed issues with current regulatory regimes, with the broad and sudden expansion in telehealth as well as deployment of health personnel to pandemic hotspots raising concerns about license to practice [40]. It also highlighted scopes of work missing in many PH systems, from vaccine regimes to community tracing [41]. The systematic review found only five articles published after the outbreak of COVID-19 in March 2020, and only two of those discussed the impact of COVID-19 on regulatory or credentialing regimes. Though only two papers in this systematic review discussed the COVID-19 pandemic, the issue of licensure and certification for the PHW has only been made more relevant in its wake. Further research on this issue is sure to be illuminating and will hopefully help drive modernization and systematization of the PHW. Some expert commentaries focusing on COVID-19 crisis and workforce-related aspects discuss forecasting the needed workforce and significant challenges for streamlining adequate credentialing processes. Additionally, retired healthcare professionals were more than welcome given the pandemic, which also brings implications to the credentialing process [42,43]. A recent Italian study on preventive medicine and resource use points to the priority of adequate and active participation of all professionals in a pandemic emergency. Such involvement and use of preventive services can potentially protect society and improve public health outcomes [44].

### Limitations

For the review process, there were several common risks and limitations associated with systematic reviews, such as selection bias and bias toward published or positive results. The professional credentialing and regulation of the PHW discussed in our systematic review appear reflected in the specialized literature published in English. The search strategy applied was broad, though the study may suffer from language bias as it only includes English language articles. By focusing only on the widely available English-language literature, we cannot reflect on the aspects of credentialing and regulations in force worldwide. In addition, we have not included official and international documents, except for a few resources on the US, UK, and New Zealand experiences. Therefore, we cannot draw conclusions about the scope of PHW authority and regulation as a whole.

Our systematic review does not focus on educational aspects or population-based analyses. The research team worked to prevent or mitigate sources of bias and completed multiple independent reviews of documents. We included gray literature and utilized multiple search engines to maximize results and reduce bias related to published results. Following screening, we critically appraised full-text materials according to the relevant JBI Critical Appraisal Tool [30]. Data extraction via a priori categories and definitions may have prevented discovering certain findings within retained materials (semantics in definitions and differences across countries). Common themes were used for aggregated findings or discourse from literature across cited materials.

Another important aspect is the field or profession. The PHW is a challenging concept and includes many occupational groups. The occupations presented in this review are not classified according to occupational standards applied on a global level (such as ISCO); instead, for this review, we have included the professions as indicated by the authors. The differences between “wider PHW” and “PH in general” introduce unnecessary deviations. Such challenges in understanding the roles, qualifications, occupations, and responsibilities within the PH field create uncertainty in the interpretation of PH delivery. Additionally, they create difficulties in generalizing credentialing and regulating the PHW on the international level.

One strong caveat of the study was that, by accommodating the differing definitions of the PHW and regulatory environments across different countries, the relevance of results may differ by audience. The analysis reveals that, despite various sources focusing on different PH professionals, most reported nursing and midwifery as a professional field of practice. There remains conflation of clinical and population-based PH services, which further complicates analyses within and across countries with respect to the PHW.

## 5. Conclusions

While the public health workforce looks substantially different across international contexts, with varying involvement in healthcare, inspection/regulation, and population-oriented activities depending on the nation in question, what appears consistent is inconsistency. Some occupations within the PHW require credentials, but not all, and it appears that within countries those credentials may vary, potentially quite a bit. Despite broad screening terms and a potential universe of approximately 3600 potential articles, only 71 meaningfully discussed credentialing and professionalization of the PHW. Despite calls from Gebbie [45], Starr [46] and others decades ago, and Czabanowska and Middleton more recently [47], considerable development must occur in this space for the field to move forward—both to more deeply describe credentials as they exist across the PHW, but also, apparently, to develop the value proposition for credentials to the PHW itself.

## Figures and Tables

**Table 1:** Occupations included within articles.

Occupation	Articles Including Occupation *
Nutrition	2
Social services	2
Dentistry	4
Not specified	2
Health education	5
Wider PH	5
Health care	14
Public health (field in general)	21
Nursing	23
**Grand total**	**78**

* Articles may have described multiple occupations.

**Table 2:** Main results.

Ref ID (Authors, Year) Country (Profession)	Summary Findings for Professional Credentialing, Certification, Licensure, Registration, and Regulation (Paraphrased)	Recommendations Extracted from Material (Paraphrased)
	**Nursing**	
0048 (Sherwood, G. D., and Shaffer, F. A., 2014) US (nursing)	*Credentialing:* is required for employment for the safety of practice. Navigation through legal and regulatory systems and the credentialing process starts with a review of the education and its compatibility with the country of practice performance.	Standardization and simplification of the credentialing process, the continuous collaboration between governments, credentialing agencies, employers, and the profession.
0052 (Kennedy, H. P., et al., 2018) US (nursing)	*Regulation:* authority maintains a register and licenses (provisional, temporary, conditional, suspended, full), defines expected standards of conduct/misconduct, and defines the scope of practice. Standards for education, clinical training, and governmental monitoring and accountability are established by *regulation* through *licensure*. Having a license offers a foundation for regulation.	Development of regulations by setting a best practice (e.g., Delphi consensus for common agreement).
0055 (Black, J., et al., 2008) Canada (nursing)	*Regulation:* conceptual framework development for competencies such as professional responsibility and accountability, self-regulation, public service, knowledge-based and ethical practice.	The development, consistency, and assessment of current competencies to clarify the roles and responsibilities (staff involvement at each stage of the process).
0077 (Pennington, K. R., Clark, K. D., and Knight, S., 2020) Australia (nursing)	*Regulation*: the regulation of health professionals and the standards for their training provide crucial and intricate protections for the public. A variety of mechanisms across Australia for suitably qualified registered health professionals in a particular area of practice.	*No clear recommendations by the authors.*
0078 (Mayra, K., Padmadas, S. S., and Matthews, Z., 2021) India (nursing)	*Regulation:* the regulatory framework includes member profiles, key definitions, membership requirements, professional registration/reregistration information and services provided, etc. Procedures are managed by various organizations, most of which do not involve nursing and midwifery representatives.	Six functions of regulatory bodies: establishing the scope of practice, pretraining registration, registration, sublicensing and maintaining authority, complaints and discipline, codes of conduct, and ethics.
0087 (Banning, M., and Stafford, M., 2008) UK (nursing)	*Regulation*: professionals can maintain their clinical governance requirements and professional competency through CPD, which benefits patients, practitioners, and organizations. CPD is a crucial component of providing high-quality clinical care.	*No clear recommendations by the authors.*
0109 (McWhirter, R., 2018) Australia (nursing)	*Regulation*: professionals must be registered according to the Nursing and Midwifery Board of Australia (NMBA) standards and policies. The NMBA is tasked with enforcing the National Law by creating registration criteria, professional codes of conduct, and other rules and regulations governing midwifery practice.	*No clear recommendations by the authors.*
0149 (Wynd, C. A., 2003) US (nursing)	*Certification*: potential benefits of certification are recognition of expertise, financial benefits, a better likelihood of advancement, employment security, and professional credibility to improve assurance in one’s expertise. Examples include certification provided to nurses which improved their qualifications as “practice professionals”.	*No clear recommendations by the authors.*
0253 (Baumann, A. et al., 2014) Canada (nursing)	*Regulation*: the Agreement on Internal Trade (AIT) AIT aims to enable regulated professionals to move freely between provinces after registration by the applicable provincial/territorial regulatory body. Self-regulation requires that the various professions establish their professional codes of conduct, licensing requirements, and practice standards.	*No clear recommendations by the authors.*
0325 (MacLaren, J., Haycock-Stuart, E., McLachlan, A., and James, C., 2016) UK (nursing)	*Regulation*: good overview of the concept of FtP. For preregistration nursing students to be deemed qualified to practice at the time of registration, they must satisfy the Nursing and Midwifery Council (NMC) requirements.	*No clear recommendations by the authors.*
0377 (Kingma, M., 2006) *Country unspecified* (nursing)	*Regulation*: when evaluating applicants’ qualifications, diplomas or degrees earned after the applicant’s initial education program are considered (resulting in the applicant’s qualifications being automatically recognized or moving on to the next step of accreditation). No perfect regulatory method, the responsibilities of regulating foreign-educated nurses are the same in each country, but various ways are used.	Regulatory agencies need to choose whether to evaluate institutions or individuals based on an approach for comparability, considerable equivalence, or full equivalence, and maintain the accuracy of their databases.
0395 (Spoel, P., and James, S., 2006) Canada (nursing)	*Regulation*: description of regulation development, competing perspectives, and, primarily, perceptions of regulations and values. Canadian midwifery movement has focused, since the early 1980s, on midwives becoming self-regulated licensed health professionals.	*No clear recommendations by the authors.*
0436 (Xu, Y., and He, F., 2012) Multiple countries * (nursing)	*Registration*, *licensure*: three categories by registration/licensure and employment status: (a) preregistration, pre-hire programs represented by Canada and what is currently in place in Australia; (b) preregistration, post-hire programs exemplified by the Overseas Nurses Program (ONP) in the UK and the newly proposed National Adaptation Program (NAP) in Australia; and (c) post-licensure, post-hire programs represented by those in the US.	Transition programs are important for language competency requirements, variations in nursing education, national health care systems, and nursing practice.
	* **Countries represented**: US, UK, Australia, Canada	
0443 (Flook, D. M., 2003) US (nursing)	*Certification, licensure:* each state willingly accepts the RN’s license to practice in the state and respects the criteria established by the nurse’s home state, including any unique restrictions. A current license, the necessary education, and evidence of experience in the specialty field are prerequisites for the certification exam. Some states resolve this issue by requiring ongoing education. The license must be renewed annually or biannually by completing a predetermined number of hours.	The nurse and the employer are accountable for maintaining competency.
0638 (Magdic, K. S., Hravnak, M., and McCartney, S., 2005) US (nursing)	*Credentialing:* all nurse practitioners who want to work in hospitals or other acute care settings must be credentialed and have their privileges reviewed periodically. For an original appointment, reappointment, and adjustment of clinical benefits, licensure is validated by the licensing board. Additionally, it occurs when a new license is issued to replace an expired one.	The acute care nurse practitioner (ACNP) role expansion in telemedicine and emergency care settings.
0667 (Oskouei, F., Nejatian, A., and Parvizy, S., 2016) Multiple countries * (nursing)	*Registration, licensure*: in certain countries, a nursing certification is necessary for registration or licensure (Diploma, Associate, Bachelor, and Master of Nursing). Passing a certification exam is needed for registration or licensure in some countries. Five years of study is required to become registered or licensed. The candidate must have good physical and mental health, pass the national exam, and have their background checked.	To deliver quality care, it is crucial to confirm nurses’ ethical, scientific, practical, and mental health.
	* **Countries represented**: Iran (primary), Canada, India, US, China, UK, Australia, Mexico, Philippines, Singapore, Japan, Thailand, Cameroon, South Korea, Poland, Peru, Russia, Armenia, Colombia, Caribbean, United Arab Emirates, Bahrain, Qatar, Oman, Saudi Arabia, Kuwait	
0867 (DiCenso, A., and Bryant-Lukosius, D., 2010) Canada (nursing)	*Regulation, certification*: administrators, clinical nurse specialists (CNSs), primary healthcare nurse practitioners (PHCNPs), and ACNPs requested standardization and national certification in response to the wide variation in educational programs across Canada and to enable more mobility for advanced practice nurses (APNs) across the country.	*No clear recommendations by the authors.*
0964 (Sheer, B., and Wong, F. K. Y., 2008) Multiple countries * (nursing)	*Regulation, certification*: an overview of nurse practitioners’ expectations in legislation, certification, and regulation in various countries. Discusses the standardization of education, regulation, and requirements to perform a profession.	*No clear recommendations by the authors.*
	* **Countries represented**: Canada, US, Latin America, Botswana, South Africa, China, Japan, South Korea, Singapore, Thailand, Australia, New Zealand, Germany, Switzerland, UK, Finland	
1039 (Wade, C. H., 2009) US (nursing)	*Certification*: the Perceived Value of Certification Tool (PVCT) assesses the perceptions of the advantages and benefits of specialty nurse certification, why nurses are certified or not, and why nurses allow their certifications to lapse.	To find evidence for the effect of certification on improved collaboration, patient outcomes, and quality of care.
1552 (North, N., Leung, W., and Lee, R., 2014) New Zealand (nursing)	*Certification*: must renew their Annual Practising Certificate (APC) each year (around their birthdays) by submitting documentation of their competency and fitness to practice.	*No clear recommendations by the authors.*
1690 (Robinson, S., and Griffiths, P., 2007) Multiple countries * (nursing)	*Credentialing, registration,*: an extensive range of various requirements for multiple countries: years of education, reception of degree, passing the exams, work expertise, post-registration program, certification in clinical specialties, further education.	*No clear recommendations by the authors.*
	* **Countries represented**: Australia, Belgium, Canada, Denmark, Finland, France, Germany, Ireland, Italy, Japan, the Netherlands, New Zealand, Norway, Spain, Sweden, Switzerland, UK, US	
4236 (Pulcini, J., Jelic, M., Gul, R., and Loke, A. Y., 2010) *Country unspecified* (nursing)	*Regulation, licensure:* to obtain preliminary data on education, licensure maintenance, and renewal, regulatory, and practice challenges facing NP-APNs as well as the appropriate political environment, the Education-Practice Subgroup of the International Nurse practitioner advanced practice Nursing Network (INP-APNN) INP-APNN prepared a pilot survey in 2006.	*No clear recommendations by the authors.*
	**Public health (field in general)**	
0017 (Polivka, B. J., and Chaudry, R. V., 2015) US (public health)	*Regulation:* accreditation as a linkage of workforce development and organizational effectiveness.	Job descriptions, competencies, guidance, and comparison on how the competencies apply to various PH positions; accreditation standards and measures.
0024 (Coen, C., and Wills, J., 2007) UK (public health)	*Regulation:* development of voluntary register (UKVR) incl. retrospective acknowledgement of skills of non-medics. Setting standards for PH specialists in the UK. Seeks to provide a clear framework enhancing career-building for the multidisciplinary public health workforce (PHW).	Addressing significant differences in the motivation and choices of the future PHW.
0027 (Vandenhouten, C. L., DeVance-Wilson, C. L., and Little, B. B., 2015) US (public health)	*Credentialing:* portfolio-based assessment. Questionnaire to investigate current certification status, motivation to gain PH certification, knowledge of exam eligibility requirements, perceived motivators and hurdles, current certification status, and benefits of credential.	*No clear recommendations by the authors.*
0029 (Evashwick, C. J., Begun, J. W., and Finnegan, J. R., 2013) US (public health)	*Certification:* to maintain certification, the certificate should be renewed every two years with documentation of ongoing education recognized by the National Board of Public Health Examiners (NBPHE), complying with US Department of Education standards.	A person who has advanced training in PH (e.g., graduate-level academic training) a PH credential, such as passing the CPH test, or training in the medical specialty of preventive medicine, is referred to as a PH professional.
0033 (Partridge, D. L. et al., 2009) US (public health)	*Credentialing:* national credentialing requires 24 hours of continuing education every 2 years. A credential for the registered sanitarian requires 30 hours of continuing education every 3 years. Includes a 40-item survey to determine the influence of credential status on competencies perceptions.	Credentialing requirements should be established and supported to encourage and institutionalize ongoing workforce training programs.
0067 (Cioffi, J. P., Lichtveld, M. Y., Thielen, L., and Miner, K., 2003) US (public health)	*Credentialing:* framework introduction. The *certification* combines performance standards for core functions in community PH practice with competencies. Individuals should accumulate a predetermined amount of continuing education credits over five years to maintain *certification*. A 190-question written test, prerequisite education, and work experience are all required for *licensure*.	Active engagement of the practice community at every level. The economic consequences of implementing certification and credentialing. An inclusive credentialing process to optimize the collaborative role that each person plays.
0074 (Leslie, K. et al., 2021) Multiple countries * (public health)	*Regulation:* leading practices from the countries by regulatory principle in definition, flexibility, accountability, efficiency, and collaboration. Variations of requirements (Canada) for endorsement for areas of practice, based on the fundamentals and covering the professional’s initial field of expertise. UK statutory organizations/regulatory authorities define a common set of core functions with substantial differences in legislation, standards, approach, and efficiency.	A clear focus on risk management to highlight flexibility of health professions’ regulation. Flexibility (allowing groups to decide on the relative responsibilities of various practitioners based on community needs) and accountability are two interrelated elements that should be balanced.
	* **Countries represented**: US, Canada, Australia, UK	
0082 (Gebbie, K. et al., 2007) US (public health)	*Credentialing* is one strategy to improve the perception of PH professionals, assist the credentialing process, and boost the general efficacy of practice. The Board has established a committee on research and evaluation to set clear goals for evaluating the value and effectiveness of the credentialing procedure.	To encourage the recognition of new PH; to extend the PHW’s perspective and support their professional and personal growth; development of a voluntary ecological approach.
0083 (Gebbie, K. M., and Turnock, B. J., 2006) US (public health)	*Credentialing:* several methods of credentialing are known, including *registration*, *certification*, and *licensure* (for doctors, nurses, and health education specialists) (for dietitians and sanitarians). Credentials show that one has mastered a particular group of skills and information through a combination of study and testing.	The pipeline techniques should be reinforced with workplace-specific approaches to fulfill the needs of the heterogeneous PH workforce. Find value in credentials and competencies by employers and health agencies.
0085 (Jehu, L. M. et al., 2018) UK (public health)	*Regulation:* introduction of a commitment to statutory regulation for all PH professionals. For those without a medical background, there is no statutory requirement to maintain registration to engage in continuing professional development (CPD).	The subject of how future PH professionals practicing for local governments will be recruited and encouraged in their development requires attention.
0090 (Hoard, M. L., and Tosatto, R. J., 2005) US (public health)	*Regulation, registration:* to create a national system of state-based registries of medical and PH volunteers, a new program (Emergency System for the Advanced Registration of Volunteer Healthcare Personnel (ESAR-VHP)) was launched.	A register may potentially act as a tool for the state’s Medical Reserve Corps (MRC) units to pre-identify and obtain credentials.
0112 (Gray, S. F., and Evans, D., 2018) UK (public health)	*Regulation*: the authority maintaining a record of professionals who have completed training and can demonstrate professional competencies has approved the final curriculum—a portfolio-type route for individuals without formal training.	Universal requirement for registration as PH specialist. Common features of the integrated training programs: curriculum and assessment standards.
0145 (Bekemeier, B., 2007) US (public health)	*Credentialing*: the intrinsic or personal benefits for the credentialed professional, such as enhanced job satisfaction, personal achievement, and progress, have mixed data supporting their value.	Barriers to credentialing such as financial burdens; individuals who contribute entirely, but do not achieve the requirements for certification receive no financial benefits.
0276 (Evans, D., and Gray, C., 2019) UK (public health)	*Regulation*: voluntarily encouraging PH practitioner registration, provided by the UK Public Health Register (UKPHR). Some practitioners have alternative registration. Recent evaluations of practitioner registration schemes show their value towards professional validation and assurance for their employers (registered professionals or those going through the registration process).	Determining whether employers see the benefits of registration in practice outweighs the costs.
0335 (Ling, K., and Belcher, P., 2014) Multiple countries * (public health)	*Regulation*: the intention is to simplify and speed up procedures, a so-called “fast-track” form of electronic documentation attesting to a professional’s qualifications and registration status, exchanged between regulatory bodies, and intended to replace the need for further paper checks.	Establish an alert system. Address how the system will work for professionals with dual registration, what information regulators will be obliged to communicate, and at what stage.
	* Countries represented: European Union, UK	
0476 (Woodhouse, L. D. et al., 2010) US (public health)	*Credentialing, certification:* The National Commission for Health Education Credentialing’s CHES certification is built on health education competencies. Best practices of certification organizations, which are relevant to individual certification, include a continuing commitment to guarantee that the competencies supporting the certification are up to date.	*No clear recommendations by the authors.*
0501 (Foster, A., 2016) US (public health)	*Credentialing, certification*: to ensure that PH graduates possess the knowledge and abilities necessary for PH practice, the CPH certificate is required. One of the essential features of a certification program is the need for ongoing professional development and education. Therefore, every 2 years, those with the CPH credential must submit 50 hours of recertification work to keep their status as active CPHs. All recertification activities must be related to the exam’s domain areas.	*No clear recommendations by the authors.*
4648 (Kurz, R. S. et al., 2017) US (public health)	*Certification:* the development of certification for the CPH. The NBPHEs set out to practice PH after acknowledging that the CPH examination should reflect the knowledge, skills, and abilities required by PH practitioners in the workplace.	*No clear recommendations by the authors.*
	**Health care**	
0096 (Saks, M., 2021) Multiple countries * (health care)	*Regulation, credentialing:* an overview of Japanese, Danish, British, Indian, Kenyan, and Brazilian professional development of professionalization and credentials for health support workers.	Cooperation and coordination through sharing of good practice, identifying the role of corporate bodies in improving global regulatory practices.
	* **Countries represented**: UK, Japan, Denmark, Brazil, India, South Africa	
0116 (Lee, S., 2008) UK (health care)	*Regulation*: after completing the necessary training and achieving a high degree of practice proficiency, approval as an accredited therapist. Before licensing a person as an expert cognitive behavioral therapist, a self-regulating organization (The British Association of Behavioural and Cognitive Psychotherapists, striving to promote excellent practice in CBT) sets its standards of training and practice. The self-regulating organization, aiming to promote good practice in cognitive behavioral therapy [CBT], determines its standards of training and practice before accrediting an individual as a proficient cognitive behavioral therapist.	Formulation of the standards for practice: the legislation and guiding principles; education and clinical supervision; processes for hiring; good practice recommendations and codes of practice; and utilizing evidence-based practice.
0279 (Hipgrave, D. B., and Hort, K., 2014) Multiple countries * (health care)	*Regulation* options for regulation: taking no action; banning/limiting dual-practice (DP); allowing DP with the regulation of behavior in public and private sectors.	Establishment and maintenance of accreditation systems by governments and professional organizations. Responsive and decentered regulation with the involvement of professional associations and civil society.
	* **Countries represented**: Australia, Bangladesh, Cambodia, China, India, Indonesia, Nepal, New Zealand, Papua New Guinea, Sri Lanka, Taiwan, Thailand, Vietnam	
0379 (Ijaz, N., and Boon, H., 2018) *Country unspecified* (health care)	*Regulation*: reflects a claim to intellectual property, first made by state actors and then by providers subject to regulation. Implementing professional regulations becomes significantly more difficult in this area’s absence of policy guidance.	WHO calls for increased statutory regulation for practitioners and practitioners of traditional and complementary medicine.
0445 (Leslie, K., 2012) Multiple countries * (health care)	*Regulation, registration*: government grants the authority to regulate, offering the profession legitimacy, autonomy, and favorable socioeconomic factors (Australia). All professions have a high degree of uniformity in registration and other regulatory processes. The concept of “fitness to practice”: the regulator (UK) initiated a separate process with possible decisions from the regulator as independent as possible.	Determine how to balance legislated consistency with autonomy for regulators. The commitment is to create umbrella legislation for consistency in regulation and possibly moving to fewer regulatory bodies.
	* **Countries represented**: Australia, UK, Canada	
0494 (Elkin, K., 2015) Multiple countries * (health care)	*Registration, certification*: certification must be achieved before one can seek registration (ability to practice), by provisional or a specialty registration. The evidence of experience can be a demonstration of clinical competence for provisional general registration.	Setting registration requirements and using those standards while making registration decisions are two ways to communicate priority.
	* **Countries represented**: Australia, New Zealand	
0513 (Gavil, A. I., and Koslov, T. I., 2016) US (health care)	*Registration, licensure*: the laws and regulations governing licensure often provide a strict “scope of practice” based on authorization to provide a list of procedures and services. This regulatory method is not adaptable enough to let the APRNs work beyond the scope of their license. The categories of licensure are based on specific occupation titles.	The process of choosing who should offer any given service incorporates competition of factors for licensure and regulation. Wait until the new approach secures statutory or regulatory approval.
0540 (Carè, J., Steel, A., and Wardle, J., 2021) Multiple countries * (health care)	*Regulation:* models of health workforce regulation vary across jurisdictions. No regulation, self-regulation, state-sanctioned self-regulation, statutory self-regulation, coregulation, and statutory regulation are some categories of occupational licensing. The regulation encourages increased regulatory cooperation between the general public, professions, and regulators, as well as a trend toward nationally based regulatory procedures.	Active participation in this policy will enable the proper regulation of T&CM within their respective jurisdictions. In addition, understanding potential enablers and impediments from a global viewpoint will help regulatory policy to be used in a variety of future contexts.
	* **Countries represented**: Australia, Canada, New Zealand, South Korea, UK, US, Egypt, Ghana, India, the Netherlands, Portugal, Saudi Arabia, Sierra Leone, South Africa, Taiwan	
0748 (Cooke, L., and Hutchinson, M., 2001) UK (health care)	*Regulation:* the claims made by regulators and assessing bodies that their evaluation procedures are thorough and transparent are unclear.	Evaluation process to determine if internationally trained practitioners have the knowledge, skills and professional qualities required to practice.
0773 (Kels, C. G., and Kels, L. H., 2013) US (health care)	*Licensing*: a review of rules for licensure in federal health care positions. Statutory support for licensure during emergencies is summarized. Practitioners typically need to hold multiple state licenses to practice telemedicine across state lines.	The introduction of telehealth and other electronic practice services will provide access to high-quality healthcare services through licensure portability.
1017 (Tang, C., and Tang, D., 2018) China (health care)	*Certification, licensing*: an approved medical school diploma, residency experience, or the successful completion of a period of practice under the supervision of a licensed physician are the minimum prerequisites for complete licensure. The National Medical Examination qualification certificate is an additional requirement. A national database containing the most recent data from each province that has authorized new doctors to practice medicine in the authority’s region.	*No clear recommendations by the authors.*
1452 (Armitage, M., and Shepherd, S., 2005) UK (health care)	*Certification, regulation*: physician assistants must pass the National Commission on Certification of Physician Assistants’ national certification exam to become certified. They must also complete 100 hours of ongoing medical education every 2 years and pass a re-certification exam every 6 years.	Tackling unresolved problems, the availability of training funding, and the potential creation of thorough curricula.
	**Wider PH**	
0028 (Ogolla, C., and Cioffi, J. P., 2007) US (Wider PH)	*Credentialing:* review found 11 studies that discussed the relationship between certification and credentialing concerning PH or health care.	Development and testing common core competencies, evaluation, communication and collaboration towards quality-of-care improvement.
0451 (Saks, M., and Allsop, J., 2007) UK (wider PH)	*Regulation, registration:* in favor of regulation for several reasons (such as the need to improve standards and consistency, to protect the public, to respond to the growing complexity of tasks, and to increase the self-esteem of support workers by acknowledging their status and skills).	Introduce a register for health support workers. Improve the management/supervision of support staff, as well as the instructions and direction provided to personnel.
0460 (Kaplan, A. D., Dominis, S., Palen, J. G., and Quain, E. E., 2013) Multiple countries * (wider PH)	*Registration, licensure:* before issuing a license, some countries require an exam; in other countries, providers are granted a license after graduating (for a fee). Physician licensing requirements are the strictest; other professionals are less likely to have active licensing processes. There are not many registration methods. Few countries can renew or update health workers’ licenses, ensuring that specific requirements are satisfied before professionals start practicing.	To strengthen licensing, regulation, and supervision, it is necessary to address the financial and human resource shortages. In addition, in countries that have traditionally solely concentrated on public sector systems, the quality assurance process will necessitate the transformation of regulations and procedures.
	* **Countries represented**: Angola, Cote d’Ivoire, Guyana, Kenya, Lesotho, Mozambique, Nigeria, Senegal, South Sudan, Caribbean, Tanzania, Uganda, Ukraine, Vietnam, Zimbabwe	
0561 (Cooper, M., Rasmussen, P., and Magarey, J., 2020) Australia (wider PH)	*Regulation*: the facilitation or restriction of qualified health practitioners (IQHPs) access to registration, migration, and employment in their trained profession in the country of destination is largely dependent on regulatory, statutory, and assessing authorities.	Strengthen the need for a re-examination and update of the existing regulatory requirements.
	**Health education**	
0005 (Taub A. et al., 2009) US (health education)	*Certification*: the development of certification for the Certified Health Education Specialist (CHES). Certification is available for individuals and aims to promote professional development and strengthen professional preparation.	Certification industry standards should be based on competencies currently used in practice.
0129 (Livingood, W. C., and Auld, M. E., 2001) US (health education)	*Credentialing*, *certification*: CHES must renew credentials annually and document 75 hours of continuing education (CE) every 5 years.	Credentialing-based competencies linked to job responsibilities; independent credentialing organization with long- and short-term funding.
0226 (Pierre Ste-Rose, S. et al., 2015) US (health education)	*Credentialing* is often not a requirement for a position. However, continuing education is required to maintain the CHES credential and is important for professional career development. In addition, other benefits contribute to the individual and collective capacity to promote community health and health equity.	*No clear recommendations by the authors.*
0473 (Taub, A., Allegrante, J. P., Barry, M. M., and Sakagami, K., 2009) Multiple countries * (health education)	*Credentialing, certification, and registration*: a continuing education requirement and a competency-based national certification exam are intended to encourage certified professionals to continue their professional growth. A registration process was created and implemented using industry standards.	*No clear recommendations by the authors.*
	* **Countries represented**: US, UK, Australia, New Zealand	
	**Dentistry**	
0388 (Johnson, P. M., 2008) Canada (dentistry)	*Regulation, credentialing, licensure:* to identify people or organizations that meet certain criteria, professional regulation procedures often involve issuing a credential. Governmental involvement is required if credentials are mandatory. Application eligibility requirements include formal education, practice standards, a registration exam, title protection, the ability to face disciplinary action, etc.	The necessary regulatory processes to ensure the profession is a cost-effective provider with a focus on quality, safety, and essential health services. The regulatory process should address the reality and future directions.
	**Nutrition**	
0060 (Landman, J. P., and Wootton, S. A., 2007) UK (nutrition)	*Regulation, registration:* discussion of a history of credentialing as a process. All applicants are required to participate in continuous education. To reapply for registration every five years, there is a (new) obligation to collect evidence (can show how they have improved their professional practice).	Regulation of non-medical health professionals; development of proficiency standards for wider PH; and a solid collective professional identity. Interprofessional and interprofessional learning.
	**Social services**	
0094 (Sugarman, M., Ezouah, P., Haywood, C., and Wennerstrom, A., 2021) US (social services)	*Certification:* different from standardized training, certification calls for an administrative authority to establish a procedure for confirming a person’s qualifications (based on skills or experience), roles or duties (incl. training, assessment, and continuing education).	Establishment of a community health worker (CHW) definition, roles, and scope of CHW practice. Determining funding and supporting leadership in workforce development.
0095 (Cornes, M., Manthorpe, J., Huxley, P., and Evans, S., 2007) UK (social services)	*Regulation, registration*: framework function by establishing standards of practice and competency, registering competent personnel, and limiting the use of particular titles to those who are registered. The concept of “fitness to practice” (FtP) is being discussed.	The regulatory framework should stimulate professionals to develop new career pathways and not lock staff into existing professional groups.
	**Articles describing multiple occupations/fields**	
0054 (Tilson, H., and Gebbie, K. M., 2004) US (public health, wider PH)	*Credentialing* (formal) by documentation and competency assurance by a formal national dialogue (led by a national commission appointed by the secretary of the US Department of Health and Human Services).	Development of voluntary certifying examination (based on the ecological model of health) and available for Master’s in Public Health (MPH) graduates.
1128 (Holmboe, E. et al., 2011) Multiple countries * (nursing, dentistry, health care)	*Regulation*: simulation-based assessment (SBA) could be used to monitor or assess practice quality.	*No clear recommendations by the authors.*
	* **Countries represented**: US, Israel, Canada	
0126 (Hilliard, T. M., and Boulton, M. L., 2012) US (public health, dentistry)	*Credentialing, certification:* an exam developed by NBPHE annually administered for PH professionals to become Certified in Public Health (CPH). Individuals who pass this credentialing exam must maintain certification through continuing education.	Increase participation in lifelong learning and improvement of competencies. Credentialing mechanisms must become widely accepted as a requirement for employment in PH.
0550 (Kerr, D. et al., 2019) US (public health, health education)	*Credentialing:* a degree and passing an exam (based on abilities created from job studies of individuals practicing the profession) are both required for obtaining individual credentials. The NCCA certification validates the CHES examination and mandates that companies uphold a legitimate and credible procedure for creating, implementing, governing, and maintaining certification programs.	*No clear recommendations by the authors.*
1128 (Holmboe, E. et al., 2011) multiple countries * (nursing, dentistry, health care)	*Regulation*: simulation-based assessment (SBA) could be used to monitor or assess practice quality.	*No clear recommendations by the authors.*
	**Profession/field not specified**	
1657 (McNair, R. P., 2005) *Country unspecified, profession not specified*	*Regulation, registration:* an acknowledgment of preregistration and regulation.	*No clear recommendations by the authors.*
0092 (Lichtveld, M. Y., and Cioffi, J. P., 2003) US (*profession not specified*)	*Credentialing, certification:* there is no national system of rewards (including certification and credentialing) to guarantee competence.	An inclusive, voluntary, competency-based certification with multiple pathways and credentialing in PH.

## Data Availability

The datasets generated and/or analyzed in the current study are available as Supplementary Content (Dataset S2).

## Supplementary Materials

The following supporting information can be downloaded at: https://www.mdpi.com/article/10.3390/ijerph20054101/s1, Protocol S1: S1_SDC_Protocol; descriptions of project methods and activities. Dataset S2: S2_SDC_Dataset; search metrics, study results, summary data.
