# Supplementary material for: A Systematic Review on Professional Regulation and Credentialing of Public Health Workforce
Source: Int J Environ Res Public Health. 2023 Feb 24;20(5):4101. doi: 10.3390/ijerph20054101 (PMC10002239; doi:10.3390/ijerph20054101)
Supplement: Supplementary file 1 [file ijerph-20-04101-s001.zip › Protocol S1.pdf]

Systematic Literature Review

Methodology

The purpose of this study is to review evidence-based literature on professional credentialing in regulation programs, standards and activities for the public health workforce (PHW).

METHODS

A. Research Questions

1. What are the most effective aspects and characteristics in identified programs (standards or activities) in professional credentialing and regulation of PHW?

2. What are common evidence-based aspects and characteristics for the performance standards to support qualified and competent PHW?

B. Search Protocol

The tool "Publish or Perish" (PoP) will be used with Google Scholar, PubMed and Web of Science to complete keyword searches across search terms. Search terms will vary across searches, but each will include variations of "public health" and "professional credentialing" and "professional regulation". The search terms included the following keywords: Public Health Workforce, Credentialing/Professional Credentialing, Regulation/Professional Regulation, Licensing, (MeSH terms). PubMed and Web of Science will return peer-reviewed literature and Google Scholar will obtain both grey and peer reviewed literature on the subjects. Search years will be 2000-2021, written in English, without country preference or preference for public health system.

1. Search Criteria

Searches to be performed using "Publish or Perish" (PoP) version 6 via keyword search to obtain results for each research question.

a. *Search Parameters*

i) **Time Period**: 2000-2021
   a) *Will add additional keystone articles beyond those found within searches.*

ii) **Language (*where available*)**: English only

iii) **Location (*where available*)**: any setting

iv) **Type of Material**
   a) ***Peer-Reviewed***: *reviews, analytic essays, summary/synthesis*
   b) ***Gray Literature***: *organizational reports, environmental scans, policy statements*

b. *Keyword Search (PoP)*

i) **Keyword List**

The main research question is split into its specific words or phrases. These topics are further split into branches which group synonymous terms

together. Some priority terms are shaded while synonymous terms are highlighted.

a) ***"workforce" (needs to be present in all):***
   (i) staff, personnel, employee, "labor force",

b) ***"public health" (strong preference for "public health" but need to expand to public health practice to attain more hits):***

c) *"Professional Regulation"*
   (i) "Regulation", "Licensing", "Registration"

d) *"Professional Credentialing"*
   (i) "Credentialing"

ii) **MeSH Terms (*did not use MeSH searching*)**

a) *Public Health*
   (i) ("Public Health"[Mesh])
   (ii) ("Public Health Practice"[Mesh])

iii) **New Terms Identified Following Searches**

a) *Not Applicable*

iv) **Exclusion Criteria (*subjective, separate from the eligibility criteria*)**

a) *Article Subject*
   (i) Article subject is within the public health education domain or undergraduate degree (regardless of if it relates to the governmental workforce and/or private workforce)
   (ii) Article subject related to PHW education and training on various levels (master and bachelor programs)
   (iii) Article subject is related to evaluations or teaching approaches in a field of PH

b) *Other*
   (i) Consider that if the material Type is "Book", it is unlikely that a quality finding may be found (not peer-reviewed, text may appear anywhere)

v) **Search Strategies (*total of 4 different searches per database*)**

a) ***Test Searches***
   (i) **Test_WoS-P-01:** **Years**: [any]; **All**: [(credentialing OR regulation OR registration OR Licensing) AND (professional) AND (workforce OR staff OR personnel OR employee OR labor force)]; **Title**: [public health,]; **Any**: [N/A]; **None**: [N/A]; **Phrase**: [N/A]

b) ***Google Scholar** (via PoP) Code: GS-01- GS-04 - 4 searches*
   (i) **GS01:** **Years**: [2000-2021]; **All**: [Public Health AND Professional AND Workforce AND ((credentialing) OR (Regulation OR Licensing OR Registration))]; **Title**: [N/A]; **Any**: [N/A]; **None**: [N/A]; **Phrase**: [N/A]
   (ii) **GS02:** **Years**: [2000-2021]; **All**: [Public Health AND Professional AND Staff AND ((credentialing) OR (Regulation OR Licensing OR Registration))]; **Title**: [N/A]; **Any**: [N/A]; **None**: [N/A]; **Phrase**: [N/A]
   (iii) **GS03**: **Years**: [2000-2021]; **All**: [Public Health AND Professional AND Personnel AND ((credentialing) OR (Regulation OR Licensing OR Registration))]; **Title**: [N/A]; **Any**: [N/A]; **None**: [N/A]; **Phrase**: [N/A]

(iv) **GS04**: **Years**: [2000-2021]; **All**: [Public Health AND Professional AND Employee AND ((credentialing) OR (Regulation OR Licensing OR Registration))]; **Title**: [N/A]; **Any**: [N/A]; **None**: [N/A]; **Phrase**: [N/A]

c) ***Web of Science*** *(via PoP) Code: WS-01- WS-04 - 4 searches*

(i) **WS01:** **Years**: [2000-2021]; **All**: [Public Health AND Professional AND Workforce AND ((credentialing) OR (Regulation OR Licensing OR Registration))]; **Title**: [N/A]; **Any**: [N/A]; **None**: [N/A]; **Phrase**: [N/A]

(ii) **WS02:** **Years**: [2000-2021]; **All**: [Public Health AND Professional AND Staff AND ((credentialing) OR (Regulation OR Licensing OR Registration))]; **Title**: [N/A]; **Any**: [N/A]; **None**: [N/A]; **Phrase**: [N/A]

(iii) **WS03**: **Years**: [2000-2021]; **All**: [Public Health AND Professional AND Personnel AND ((credentialing) OR (Regulation OR Licensing OR Registration))]; **Title**: [N/A]; **Any**: [N/A]; **None**: [N/A]; **Phrase**: [N/A]

(iv) **WS04**: **Years**: [2000-2021]; **All**: [Public Health AND Professional AND Employee AND ((credentialing) OR (Regulation OR Licensing OR Registration))]; **Title**: [N/A]; **Any**: [N/A]; **None**: [N/A]; **Phrase**: [N/A]

d) ***PubMe****d (via PoP) Code: PM-01- PM-04 - 4 searches*

(i) **PM01:** **Years**: [2000-2021]; **All**: [Public Health AND Professional AND Workforce AND ((credentialing) OR (Regulation OR Licensing OR Registration))]; **Title**: [N/A]; **Any**: [N/A]; **None**: [N/A]; **Phrase**: [N/A]

(ii) **PM02:** **Years**: [2000-2021]; **All**: [Public Health AND Professional AND Staff AND ((credentialing) OR (Regulation OR Licensing OR Registration))]; **Title**: [N/A]; **Any**: [N/A]; **None**: [N/A]; **Phrase**: [N/A]

(iii) **PM03**: **Years**: [2000-2021]; **All**: [Public Health AND Professional AND Personnel AND ((credentialing) OR (Regulation OR Licensing OR Registration))]; **Title**: [N/A]; **Any**: [N/A]; **None**: [N/A]; **Phrase**: [N/A]

(iv) **PM04**: **Years**: [2000-2021]; **All**: [Public Health AND Professional AND Employee AND ((credentialing) OR (Regulation OR Licensing OR Registration))]; **Title**: [N/A]; **Any**: [N/A]; **None**: [N/A]; **Phrase**: [N/A]

C. Review Protocol

Citations will first undergo a preliminary relevance assessment (title and/or abstract against the research question for eligibility and according to several criteria such as the field of PH performance; articles discussing professional credentialing or regulation or licensing or registration (at least one aspect); articles focusing on PH graduates (PH wider workforce) working a field of PH. Two reviewers plus one to resolve conflicts. Full-text articles will be obtained for relevant citations. Full-text articles will then be appraised for eligibility according to the 10 criteria of JBI Critical Appraisal Tool for Qualitative Research. Two reviewers plus one to resolve conflicts. Filling in PRISMA Flow Diagram to ensure adequate flow of information through the different phases of a systematic review.

Data extraction will utilize a spreadsheet with the following extraction columns such as Standard Elements (Author, year, journal, etc.; eligibility criteria for abstract screening; the 10 criteria of JBI Critical Appraisal Tool for Qualitative Research; Quotes; Other Notes.

1. Retrieving Citations and Screening (including PRISMA Flow Diagram)

   a. ***Reviewers (1–3)***

i) **OG**: relevance screening; eligibility review; data extraction/synthesis/analysis; drafting

ii) **JO**: relevance screening; eligibility conflict resolution; data extraction/synthesis/analysis; drafting

iii) **KC:** conflict resolution; eligibility review; data extraction/synthesis/analysis; drafting

b. ***Document Search*** *(according to above)*

i) **Remove** any duplicates

c. ***Relevance Assessment*** *(title and/or abstract against research question)*

i) **Retain** if appropriate – *retrieve full article*

ii) **Remove** if inappropriate – *document why removed upon disagreement*

2. **Critical Appraisal of Evidence** (including PRISMA Flow Diagram)

a. ***Eligibility Appraisal*** *(abstracts). Reviewers: OG, JO, AV, KP*

i) **Retain** if meeting eligibility criteria mentioned above

ii) **Remove** if not meeting criteria – *document which criteria may be missing*

b. ***Quality Appraisal of Documents*** *(full article)**.** Reviewers: OG, JO, AV, KP*

i) **Retain** if meeting eligibility criteria according to the 10 criteria of JBI Critical Appraisal Tool for Qualitative Research

ii) **Remove** if not meeting criteria – *document which criteria may be missing*

3. **Data Collection Process**

a. ***Data Extraction*** *(spreadsheet)*

b. ***Analysis Categories for Theming*** *(spreadsheet)*

D. Risk of Bias Considerations

This review will consider peer reviewed literature and gray/white literature. Titles and abstracts (screening) will be reviewed by 2 researchers independently, with 1 resolving screening disagreements as needed. Full-text articles (eligibility) will be reviewed by 2 researchers independently, with 1 resolving eligibility disagreements as needed.

Team (7 researchers) anticipates that in the systematic review the team will 1) screen for eligibility criteria and 2) makes use of quality appraisal according to the criteria of JBI Critical Appraisal Tool for Qualitative Research

E. Data Synthesis and Analysis

We will include all eligible studies and records which meet our inclusion criteria within a specified time frame according to the PRISMA statement. After reaching the consensus on the included material we will perform key theming of the included evidence. Several example categories are country of performance; the field of PH (organization or profession); evidence-based approach, the methodological background of the process/performance standards; transferability; etc. The set of the final categories will be determined after understanding the content of the results. Additionally, we will apply frequency analyses and use descriptive statistics to summarize several categories (or measures). In our systematic review, we will not be

focusing on testing the hypotheses or population-based analyses, therefore we will not perform inferential statistical analysis.

The team will be examining the evidence related to professional credentialing and professional regulation (including professional registration and licensing). Therefore, reaching the consensus on the included material we will perform key theming of the included evidence and separately analyze those components of professional regulation ("registration" and "licensing"). We will apply descriptive statistics the key theming of the abovementioned sub-categories.
